# Analysis of Treatment of 3 Patients with Acute-on-Chronic Liver Failure

**DOI:** 10.1155/2018/7421502

**Published:** 2018-11-22

**Authors:** Jun Lei, Yi-lan Zeng, Lv-ye Xu, Ying Wang

**Affiliations:** ^1^Department of Infectious Disease, General Hospital of the Yangtze River Shipping, Wuhan Brain Hospital, Wuhan 430010, China; ^2^Department of Hepatitis, Public Health Clinical Center of Chengdu, Chengdu 610066, China

## Abstract

Acute-on-chronic liver failure (ACLF) is an acute liver decompensation that occurs within 4 weeks on the basis of chronic liver disease. At present, the treatments of ACLF include general supportive treatment, etiological treatment, prevention and treatment of complications, artificial liver treatment, and liver transplantation. Many studies suggest that stem cell therapy may become a new treatment for patients with ACLF. Our department has also tried the application of this treatment. Now, there are three cases of stem cell therapy for patients with ACLF by our department which will be briefly reported.

## 1. Introduction

Liver failure is a severe liver damage caused by a variety of reasons, resulting in serious disorders or decompensation of its synthesis, detoxification, excretion, biotransformation, and other functions, which leads to a group of clinical syndromes including coagulation disorders, jaundice, hepatic encephalopathy, and ascites. Liver failure is featured with rapid progression, a lot of complications, and high mortality. While acute-on-chronic liver failure (ACLF) is an acute liver decompensation that occurs within 4 weeks on the basis of chronic liver disease. Patients may experience a series of clinical syndromes such as jaundice, hepatic encephalopathy, and coagulopathy, and the mortality rate is more than 60% [1]. At present, the treatments of ACLF include general supportive treatment, etiological treatment, prevention and treatment of complications, artificial liver treatment, and liver transplantation. Based on the above situation, patients with ACLF face not only severe illness and rapid progression [2] but also unsatisfactory effect of existing treatments, leading to high mortality. In recent years, stem cell therapy is a new way to treat liver failure, and there are three cases of stem cell therapy for patients with ACLF by our department which will be briefly reported.

## 2. Case 1

Male, 48 years old, with a history of chronic hepatitis B, had been treated with entecavir for antiviral therapy for two years, which has been stopped without doctors' guidance for five months till now. Two weeks ago, the patient gradually suffered from fatigue, abdominal distension, yellow urine, and eye irritation and was diagnosed as liver dysfunction in the local hospital. For further diagnosis and treatment, the patient was admitted to our hospital on September 14, 2012. Liver function tests before hospitalization showed alanine aminotransferase (ALT) 1007 U/L, aspartate transaminase (AST) 864 U/L, total bilirubin (TBIL) 218.7 *μ*mol/L, and direct bilirubin (DBIL) 171.7 *μ*mol/L, while hepatitis B virus markers showed HBsAg+, HBeAg+, HBcAb+, and HBV-DNA 3.21 × 10^6^ IU/L. According to the patient's conditions, he was given a variety of treatments, involving conventional liver protection, reducing enzyme activity, eliminating jaundice, and entecavir antiviral therapy. With the consent of the patient, he was treated with hepatic arterial infusion of the umbilical cord blood stem cells (UC-MSCs mononuclear cells 42.4 × 10^9^/ml, flow cytometry CD34+ and CD33+ stem cells 8.9 × 10^6^/ml with a total input volume of 40 ml). The relevant tests were performed regularly after the infusion, including ALT, ALB, TBIL, and PTA. The patient was discharged from our hospital with a better health condition on October 26, 2012, and went back to the local hospital for continual treatment. Currently, the follow-up status is good. The changes of the patient's indicators during treatment are shown in Table 1.

## 3. Case 2

Male, 54 years old, had a history of daily drinking with the amount of 100–150 g per day for 30 years, been diagnosed as “alcoholic cirrhosis” in other hospital one year ago. The patient began to feel the abdominal distension, accompanied by fatigue, yellow urine, and jaundice, one month ago. On April 11, 2012, the patient started the treatment in our hospital. After admission, the tests revealed ALT 924.6 U/L, AST 817.3 U/L, TBIL 274.2 *μ*mol/L, and DBIL 189.4 *μ*mol/L. With the consent of the patient, he was treated with peripheral injection of the umbilical blood stem cells (UC-MSCs mononuclear cells 39.6 × 10^9^/ml, flow cytometry CD34+ and CD33+ stem cells 10.1 × 10^6^/ml with a total input volume of 40 ml). The relevant tests were performed regularly after the infusion, including ALT, ALB, TBIL, and PTA. At the same time, the patient was given supportive treatments such as stopping drinking, conventional liver protection, reducing transaminase, and eliminating jaundice as well as albumin treatment. The input of stem cells promotes the regeneration of liver cells and the recovery of various physiological functions. The changes of the patient's indicators during treatment can be seen in Table 2. The patient was discharged with a better health condition on May 21, 2012, and regular follow-up was conducted after discharge.

## 4. Case 3

Male, 52 years old, had a history of hepatitis B for more than 10 years without regular treatments. The patient often drinks alcohol, and the average amount is around 250 g each time. The patient started to feel malaise and fatigue, abdominal distension on January 12, 2013, but the symptoms were largely ignored at that time. Five days ago, after drinking 250–300 g of alcohol, the patient suffered from significantly increased fatigue, together with poor appetite, yellow urine, and jaundice. The patient started the treatment in our hospital on January 19, 2013. Outpatient tests for liver function showed ALT 771 U/L, AST 608 U/L, TBIL 176.5 *μ*mol/L, and DBIL 102.7 *μ*mol/L, while hepatitis B virus markers showed HBsAg+, HBeAb+, HBcAb+, and HBV-DNA 1.86 × 10^6^ IU/L. After admission, the patient received a series of treatments, including entecavir antihepatitis B virus therapy, liver protection, eliminating jaundice and reducing enzyme activity treatment, immune regulation, and complication prevention. The review of liver function on January 28, 2013, indicated ALT 465 U/L, AST 504 U/L, and TBIL 239.5 *μ*mol/L, showing the rapid progression of disease. With the consent of the patient, he was treated with peripheral injection of the umbilical blood stem cells (UC-MSCs mononuclear cells 37.4 × 10^9^/ml, flow cytometry CD34+ and CD33+ stem cells 8.9 × 10^6^/ml with a total input volume of 40 ml). The relevant tests were performed regularly after the infusion, including ALT, ALB, TBIL, and PTA. The reexamination for liver function on February 7, 2013, showed ALT 307 U/L, AST 446 U/L, and TBIL 265.3 *μ*mol/L, which indicated that the disease progression was significantly advanced. On February 12, the review of liver function revealed ALT 265 U/L, AST 372 U/L, and TBIL 308.1 *μ*mol/L. With the progression of the patient's health condition, the follow-up treatment was conducted. The patient was given bilirubin adsorption treatment for 2 times, while the effect is still poor, and the family abandoned the treatment and discharged (Table 3).

## 5. Discussion

In our country, hepatitis B virus (HBV) infection is the main cause of acute-on-chronic liver failure (ACLF) [3], the mortality of which is as high as 62.18%–72.3% [4–6]. Other causes of ACLF include alcohol, drugs and toxic substances, and genetic metabolism. The main treatments include comprehensive medical treatment, artificial liver treatment, and liver transplantation. Comprehensive medical treatment covers supportive treatment, etiological treatment, prevention and treatment of complications, and the use of other medications, the curative effect of which is limited. Orthotopic liver transplantation serves as the only effective treatment, but its application is limited due to donor liver deficiency, rejection after transplantation, and adverse effects caused by the use of a great many immunosuppressants after transplantation. The artificial liver support system can not only remove all kinds of toxic substances stranded in the body caused by liver damage but also replenish some bioactive substances, so as to maintain the stability of the inner environment of the body, gain time for hepatocyte regeneration, and reduce the patients' mortality [7]. However, studies by Li [8] (artificial liver) have shown that plasma exchange is more effective for the clinical treatment of early and intermediate stage liver failure, while its effect is relatively poor for advanced liver failure. Therefore, there is an urgent need to find a way to treat and prolong the survival time of ACLF patients.

Stem cell transplantation can provide a large number of hepatocyte-like cells, replacing the synthetic and metabolic function required by the normal liver, and change the tissue microenvironment through a paracrine mechanism [9]. Therefore, many studies suggest that stem cell therapy may become a new treatment for patients with advanced liver disease [10], especially umbilical cord mesenchymal stem cells (UC-MSCs) have been a hot research topic in recent years. Studies from Li et al. indicate that, for patients with ACLF, UC-MSCs treatment is beneficial to the recovery of patients' liver function and can effectively improve the survival rate of patients [11].

At present, there is no clear mechanism of stem cell to improve liver function and mainly the following two hypotheses [12–14]: first, the differentiation of stem cells. Under the action of microenvironment, stem cells can differentiate into functional liver cells. Second, the role of stem cells in paracrine secretion, which can promote the multiplication capacity of endogenous hepatocytes. Based on the two hypotheses and a large number of previous clinical studies, the efficacy and safety of stem cells in the treatment of end-stage liver disease have been confirmed.

All three patients in this study had received an input of umbilical cord blood mesenchymal stem cells (UC-MSCs), but the etiology and the method of input are different: Case 1, patients for HBV-related ACLF, given the hepatic artery interventional infusion; Case 2, patients for alcohol-related ACLF, given peripheral venous infusion; and Case 3, patients for hepatitis B combined with alcohol-related ACLF, also given peripheral intravenous infusion. According to the treatment results of 3 patients, Case 2 patient with ACLF caused by alcohol alone had the best therapeutic effect, and the hepatic artery intervention infusion had the fastest effect and the best curative effect, but which need a large sample of clinical trials to confirm. And further clinical observation and analysis are needed for long-term prognosis and long-term safety after UC-MSCs transplantation.

The immune mechanism of liver failure is not yet clear, and it requires further study on the immune manifestations of different causes, different stages, and different patients. For patients with ACLF, no treatment is a panacea. The most appropriate treatment can only be achieved through targeting the right patient, choosing the right medicine, and seizing the right time. It is believed that, with the deepening of research on liver failure, the correct answer will be found in the complicated pathophysiological mechanisms and clinical manifestations, so that clinicians can make timely and correct judgment and treatment options for patients in order to obtain the maximum clinical benefit.

## Figures and Tables

**Table 1:** The changes of the patient's indicators during treatment (Case 1).

	ALT (U/L)	ALB (g/L)	TBIL (*μ*mol/L)	PTA (%)	MELD score
0 week	1007	31.4	218.7	36	25
2nd week (infusion of cord blood stem cells)	652	32.0	201.8	39	24
4th week	108.1	32.7	147.5	47	22
8th week	79.4	33.2	105.2	54	18
12th week	39.6	32.9	88.3	57	14

**Table 2:** The changes of the patient's indicators during treatment (Case 2).

	ALT (U/L)	ALB (g/L)	TBIL (*μ*mol/L)	PTA (%)	MELD score
April 11, admission	924.6	228.6	274.2	38	29
April 28, before the stem cell inputted	239.6	29.4	279.3	39	29
May 6, after one week of the stem cell inputted	97.4	30.8	201.7	41	27
May 21, discharge	79.7	31.9	125.9	43	17
June 18, one month after discharge	43.9	34.7	76.3	46	13

**Table 3:** The changes of the patient's indicators during treatment (Case 3).

	ALT (U/L)	ALB (g/L)	TBIL (*μ*mol/L)	PTA (%)	MELD score
January 19, admission	771	31.8	176.5	42	22
January 28, before the stem cell inputted	465	30.2	239.5	40	25
February 7, after one week of the stem cell inputted	307	29.5	265.3	40	28
February 12	265	28.3	308.1	36	29
March 1	87	25.4	351.2	31	32
